# A defective ground technique for reducing crosstalk induced by on-chip antennas in RFIC environment

**DOI:** 10.1038/s41598-026-52340-x

**Published:** 2026-05-12

**Authors:** Vipin Singh, Harshavardhan Singh, Mohammad Alibakhshikenari, Patrizia Livreri

**Affiliations:** 1 Department of Electronics and Communication Engineering, Amrita School of Engineering, Amrita Vishwa Vidyapeetham, Coimbatore, India; 2 Lero, The Research Ireland Centre for Software, College of Science and Engineering, School of Computer Science, University of Galway, Galway, H91 TK33 Ireland; 3 Department of Electrical and Electronics Engineering, Dogus University, 34775 Umraniye, Istanbul, Türkiye; 4 Department of Engineering, University of Palermo, 90128 Palermo, Italy

**Keywords:** Crosstalk, On-chip antenna, Miniaturization, Defected ground structure, Radio frequency integrated circuit, Engineering, Materials science

## Abstract

Crosstalk is induced due to the on-chip antenna (OCA) in RFIC, which affects nearby RFIC blocks, degrading signal integrity and overall system performance. In this work, a defected ground structure (DGS) based technique with a meandered loop OCA is proposed to reduce crosstalk at a frequency of 10.3 GHz. The DGS is introduced between the silicon substrate and the top metal layer meandered loop OCA, separated by a thick $SiO_2$ layer, which effectively reduces the crosstalk. Moreover, to make the study suitable for compact ICs, crosstalk analysis is performed by keeping the blocks at various positions with respect to the reference antenna separated at $d = 6$ mm with orientations of $0^\circ$, $45^\circ$, and $90^\circ$ with respect to the x-axis. The obtained $S_{12}$ values reach up to $-85$ dB, $-91$ dB, and $-77$ dB, respectively. Furthermore, an extensive parametric analysis is performed to justify the design considerations and optimum values of the parameters. To examine the crosstalk behaviour of the proposed OCA, an equivalent circuit model analysis is performed. The characterization is validated using a fabricated prototype. The results demonstrate that the proposed multi technique based multi-path suppression of electromagnetic coupling method provides ultra-high isolation, compact size, and an improved figure of merit for minimizing crosstalk in highly integrated RFIC environments.

## Introduction

The demand for highly integrated and small-size wireless radio-frequency integrated circuits (RFICs) has accelerated over the last 10 years with the growing demands of Internet of Things (IoT) applications^1^, wearable^2^, wireless sensors^3^, 5G and 6G communication systems^4^. The seamless integration of all RF building blocks onto a single chip, as realized in System-on-Chip (SoC) architectures, constitutes a key objective in modern RFIC design, enabling enhanced miniaturization and higher system-level integration efficiency^5^. Although circuit devices like power amplifiers (PAs)^6^, low-noise amplifiers (LNAs)^7^, mixers^8^, bandpass filters^9^ and voltage-controlled oscillators (VCOs)^10^ have been successfully integrated through advanced CMOS and SiGe processes, the antenna on chip has continued to be a major bottleneck.

Moreover, at lower microwave frequencies, particularly below 30 GHz, the corresponding increase in wavelength (in the order of several millimeters to centimeters) results in substantially larger antenna dimensions, posing significant challenges for seamless on-chip integration within compact RFIC architecture. This makes conventional off-chip antennas the default choice, resulting in additional packaging complexity and signal losses at the chip–package interface.

To address this challenge, on-chip antennas (OCAs) have emerged as a potential solution, enabling seamless integration with RFICs. Over the past two decades, numerous studies have been reported on the design, optimization, and miniaturization of OCAs^11,12^. These reports show that OCAs can minimize packaging cost, enhance compactness, and enable entirely integrated system-on-chip (SoC) solutions.

In spite of their benefits, OCAs have serious performance constraints that also limit their applications. One of the biggest issues is their inherently low radiation efficiency and gain due to substrate losses, surface waves, and limitations of size^13^. As a consequence, much research has gone into overcoming the OCA performance using mechanisms like substrate engineering, slotting, metamaterial loading, and array-based configurations^14,15^. Although these techniques have been promising, the pervasive use of OCAs in commercial RFICs has so far been limited due to one of the equally critical hurdles, i.e., electromagnetic coupling induced by OCAs. Crosstalk is unwanted electromagnetic coupling between the OCA and nearby circuit blocks on the chip. In a deeply integrated RFIC environment, sensitive circuit blocks may be seriously affected by such coupling. Moreover, the performance of OCAs is also affected due to the presence of these other blocks of RFIC^12^. This could lead to compromised noise performance, decreased linearity, frequency instability, or even failure of key circuit blocks. Several crosstalk reduction techniques have been reported in the literature, including the use of high-resistivity substrates^16^, isolation using through-silicon vias (TSV)^17,18^, guard rings^19^, and other mitigation methods^20,21^. However, the influence of crosstalk on the performance of other blocks of RFIC has been relatively less reported. As a consequence, a large gap has remained in the implementation of OCA in the real system-level integration.

This study primarily deals with the issue of crosstalk caused by OCAs, which affects the performance of other blocks in RFICs. These blocks can be any non-radiating (such as inductor, LNA, PAs, etc) or radiating structure (i.e., antenna). To examine the worst-case scenario inside the RFIC environment, the interacting adjacent block is also considered as an identical OCA, allowing for a complete analysis of antenna-to-antenna crosstalk in the same chip environment. Additionally, the research examines the impact of different antenna positions on the level of crosstalk. Moreover, as it is proven that a Defective Ground Structures (DGS) has been successfully applied to improve or reduce the coupling between the components^22,23^, a DGS based technique is explored in the proposed study to reduce the crosstalk in the RFIC environment. Furthermore, this research offers practical design insights that can help inform crosstalk-aware OCA integration and facilitate future compact RFIC systems that are more reliable and robust.

The remaining paper is organised as follows: Section I discusses design considerations of the proposed meandered loop OCA. Section II presents the parametric analysis to enable the optimum design and study the variation in reflection and radiation parameters under various conditions. Section III shows the equivalent model for crosstalk analysis, which highlights the mathematical aspect to reduce crosstalk between two adjacent blocks. Section IV demonstrates the obtained result and its analysis in detail. Section V highlights the comparison of the proposed study with previously reported literature. Finally, the key findings and insights of crosstalk reduction in OCA technology, with particular emphasis on its role in next-generation communication systems, are concluded in Section VI.

## Antenna design

**Fig. 1:**
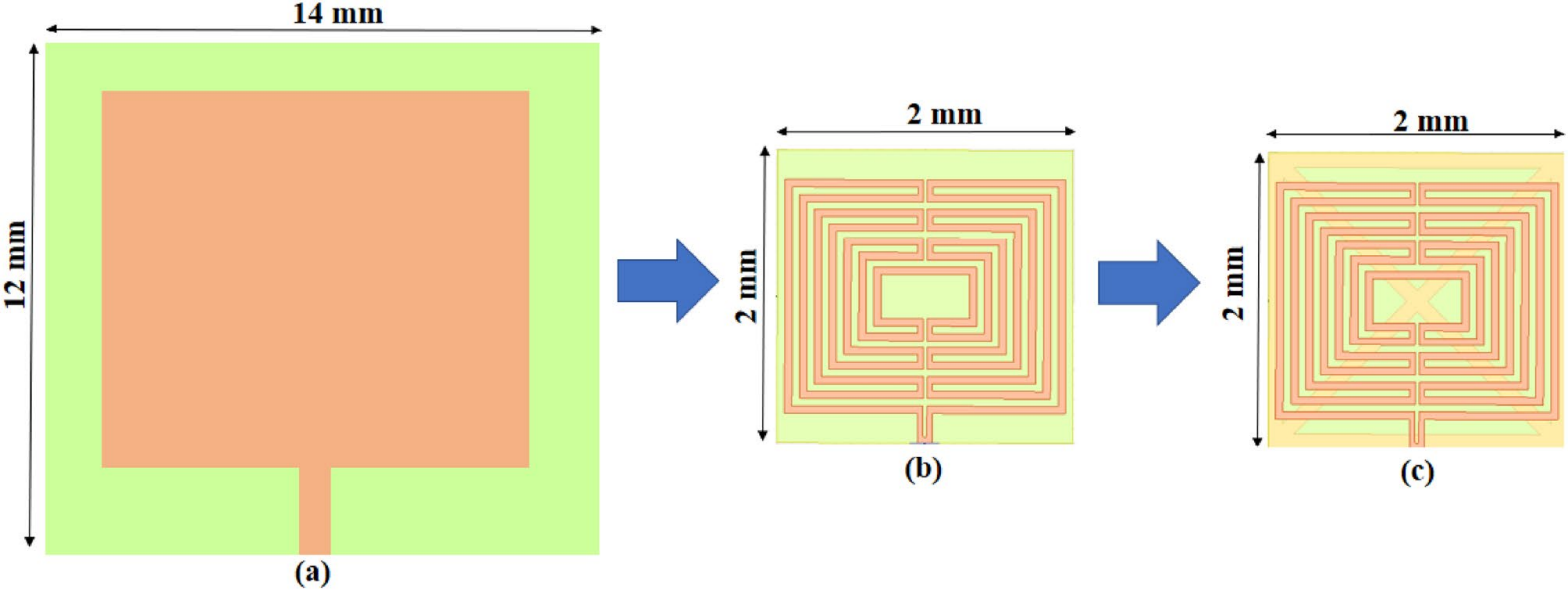
Step-wise antenna design approach: (**a**) Simple Patch; (**b**) Patch + Meandered loop; (**c**) Patch + Meandered loop + DGS.

###  Design consideration

The step-wise design process of the OCA is shown in Fig. 1a–c. The design process begins with an integrated patch antenna suitable for the RFIC environment. The designed model is simulated by ANSYS HFSS. Radiation boundary conditions are used at a distance of 5 mm from the structure. This ensures the simulation occurs under open space conditions and prevents any reflection effects. The simulation process uses the adaptive meshing method, allowing the design to be accurately simulated. The condition for converging the adaptive meshing is when the maximum Delta-S ($\Delta$S) value becomes less than or equal to 0.02, which indicates a difference of no more than 2% in each iteration process. A maximum of 20 adaptive passes is implemented to obtain stable and reliable results.

**Fig. 2:**
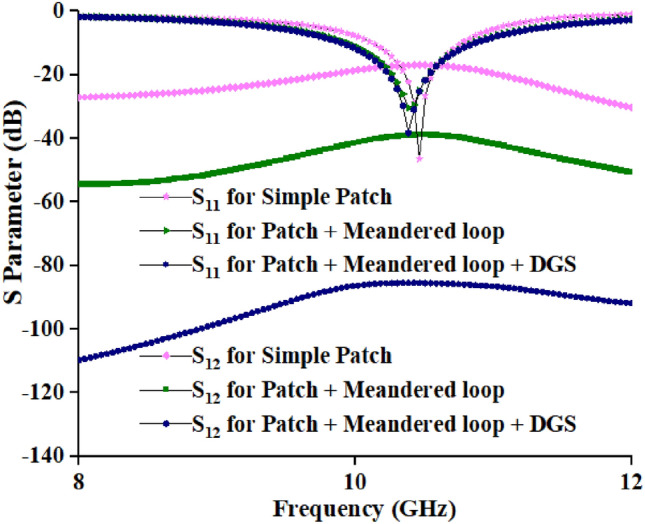
$S_{{11}} and S_{{12}}$ for (**a**) Simple Patch (**b**) Patch + Meandered loop (**c**) Patch + Meandered loop + DGS.

The applications of RFIC and its front-end components are extensively explored in the design of Radars^24^, Phased array systems^25^ Power amplifier^26^ and LNA^27^ at X-band and motivated for selecting it as the operating frequency band for the proposed work. The antenna is implemented using standard 180 nm Si based CMOS technology, which typically includes 9 to 11 metal layers sandwiched between layers of silicon dioxide ($SiO_2$). In this design, the topmost metal layer is utilized for the antenna structure to maximize radiation efficiency toward the air and minimize substrate coupling. The patch antenna designed on the Si substrate has the physical dimensions of 14 $\times$ 12 mm^2^ as shown in Fig. 1a. The designed OCA achieves a resonant frequency of 10.5 GHz and demonstrates good 50 $\Omega$ impedance matching, quantified by an $S_{11}$ of − 46 dB^28^. To address chip-area constraints, antenna miniaturization is performed using the meandered loop technique. Fig. 1b reflects that the meandering technique effectively extended the electrical length of the OCA without increasing its physical dimensions, thereby reducing the antenna footprint to 2 $\times$ 2 mm^2^, achieving more than 85% reduction in size. The $S_{11}$ for the patch, meandered loop, and meandered loop with DGS are shown in Fig. 2, where both the meandered loop and the meandered loop with DGS operate at 10.3 GHz. Fig. 2 also displays that after miniaturization, the crosstalk ($S_{12}$) is reduced up to − 38 dB from − 17 dB. To reduce it furthermore, a DGS is incorporated into the ground of OCA as illustrated in Fig. 1c. The incorporation of the DGS enhances the radiation performance of OCA, which is drastically reduced due to a significant reduction in the aperture of the antenna^29^. Moreover, the DGS also reduces the crosstalk level up to − 85 dB. It is because DGS forms a resonant slot in the ground plane that interrupts the continuous flow of shield currents, thereby reducing the common current paths responsible for mutual coupling between adjacent RFIC blocks^23^.

**Fig. 3:**
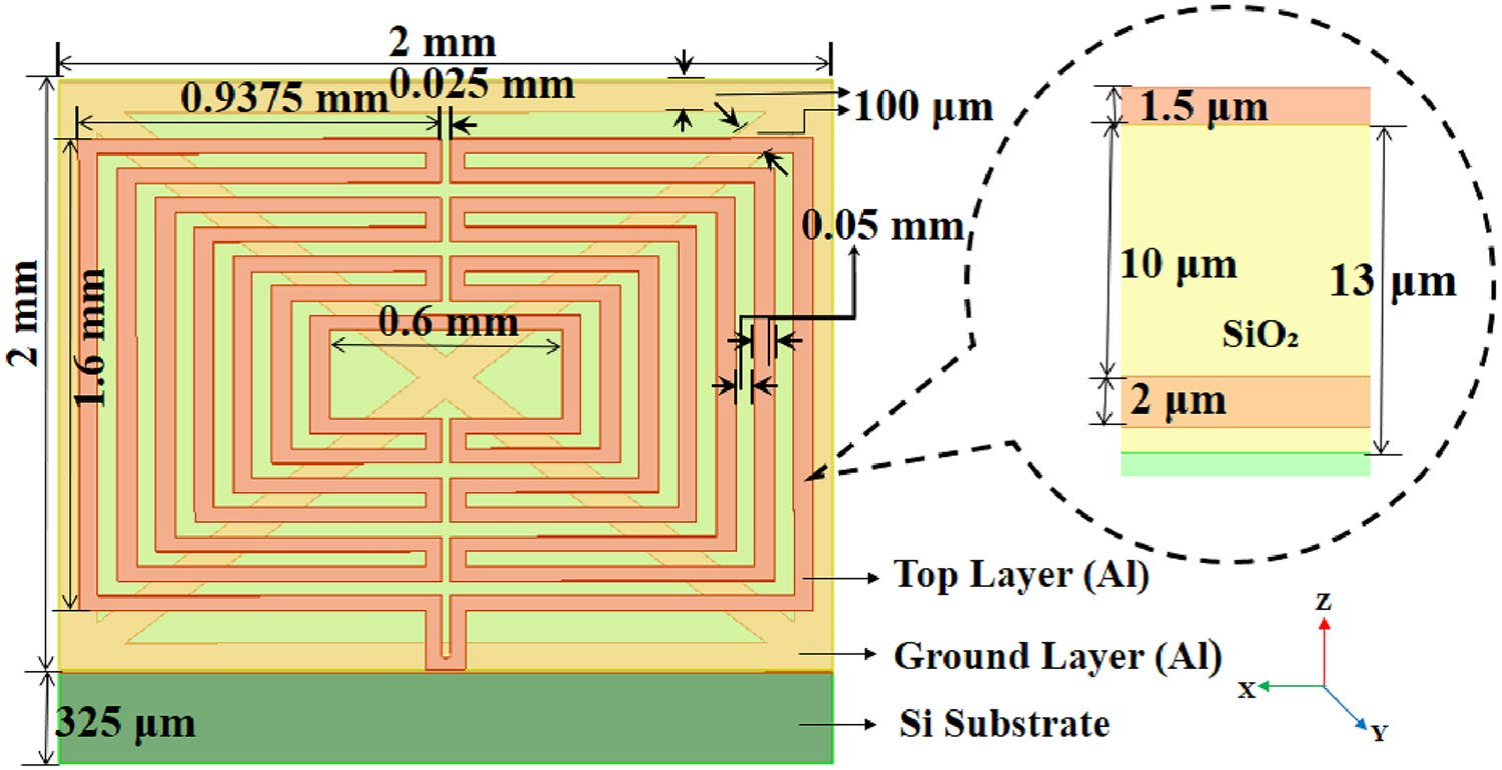
3D view of On-chip Antenna structure with cross-sectional view (in inset).

**Fig. 4:**
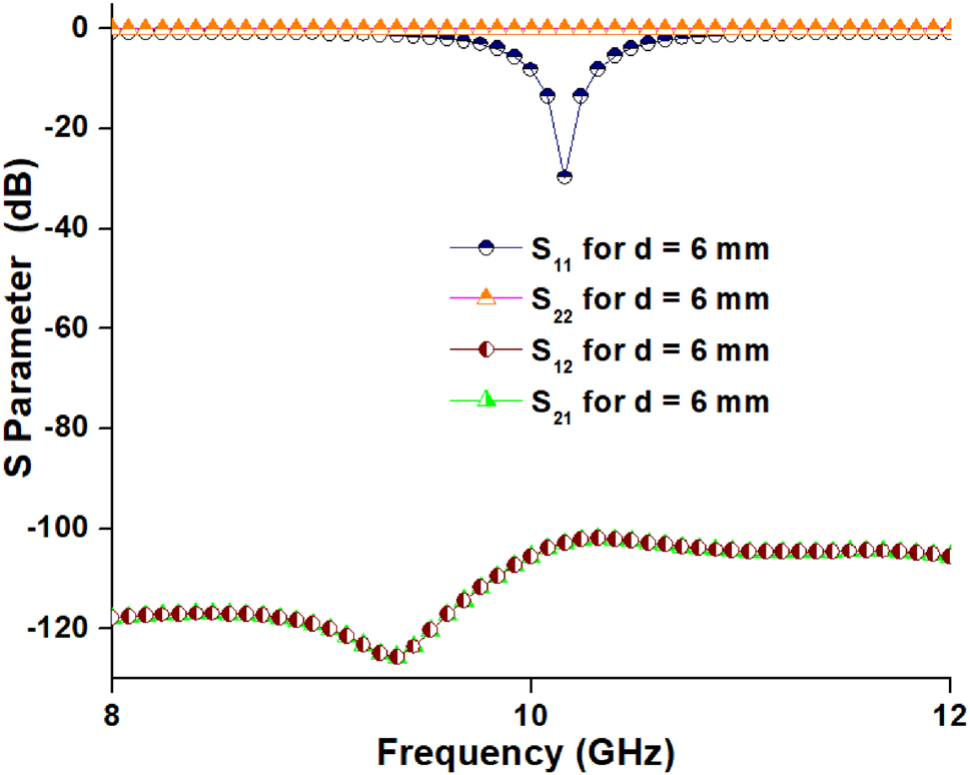
S parameters analysis, when other block is Non-radiating and placed at ‘d’ = 6 mm at 0°from x-axis.

Furthermore, to clearly distinguish between different operating scenarios, the best-case crosstalk corresponds to the condition in which the second block remains in a non-radiating state, whereas the worst-case crosstalk occurs when the second block is actively radiating (i.e., an identical OCA). As illustrated in the Fig.4, the non-radiating condition of the second block is verified by the $S_{11}$ response. Under this configuration, the $S_{12}$ level is observed to be well below − 102 dB at the resonance frequency when the second block is orientated at 0°with respect to the x-axis. Owing to the extremely low crosstalk achieved in this case, other angular orientations for the best-case scenario are not further considered in this study.

### Meandered loop OCA design

The proposed meandered loop OCA of 2 $\times$ 2 mm^2^ is designed on a 325 μm thick Si substrate as shown in Fig. 3. Si is inherently not an ideal substrate for antenna design as it has low-resistivity ($\rho$ = 5 $\Omega$.cm) and has a high dielectric constant ($\epsilon _r = 11.9$). As a result, a significant portion of the radiated signal power is absorbed into the substrate rather than radiated into the air, leading to substantial substrate loss. However, Si wafers are chosen for fabrication due to their wide availability and global acceptance in IC manufacturing foundries while remaining economical. Here, a layer of 13 μm thick $SiO_2$ is placed on the wafer. This thick layer functions as an insulating medium, effectively suppressing radiation leakage into the substrate and thereby improving the overall radiation efficiency of the antenna. At the top, a 1.5 μm Al layer is deposited as a metal layer for designing a meandered loop antenna. Now, DGS with two orthogonal crossed bow-tie shaped slots is implemented in one of the intermediate Al layers of the CMOS layout. The crossed bow-tie DGS geometry is chosen considering both radiation behaviour and CMOS fabrication constraints. Since antenna radiation is dominant at the edges, the ground plane beneath these regions is kept intact, while the central portion is etched to suppress surface currents and reduce crosstalk. Additionally, CMOS metal density rules (30 to 70% metal retention) restrict arbitrary etching, and the proposed shape satisfies these constraints while providing effective isolation. The dimensions were optimized through parametric simulations.

Moreover, a coplanar waveguide (CPW) technique is used to feed the OCA. The CPW lines are excited using a Ground Signal Ground (GSG) probe, which is briefly discussed in^30,31^. To ensure the successful connection between the antenna and probes, the optimized ground pads have been created on the same plane as the antenna (not shown in top view). The proposed OCA operates at a resonant frequency of 10.3 GHz and achieves $S_{11}$ of − 38 dB, indicating excellent impedance matching and minimal signal reflection as demonstrated in Fig. 2.

**Fig. 5:**
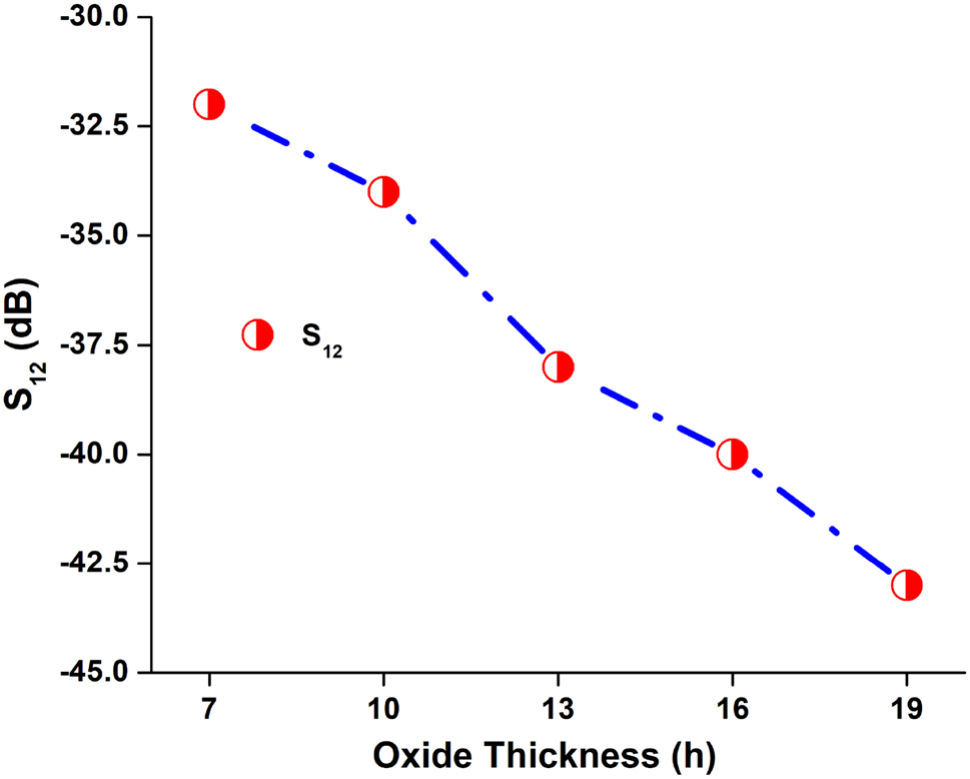
$S_{12}$ versus oxide thickness without DGS.

**Fig. 6:**
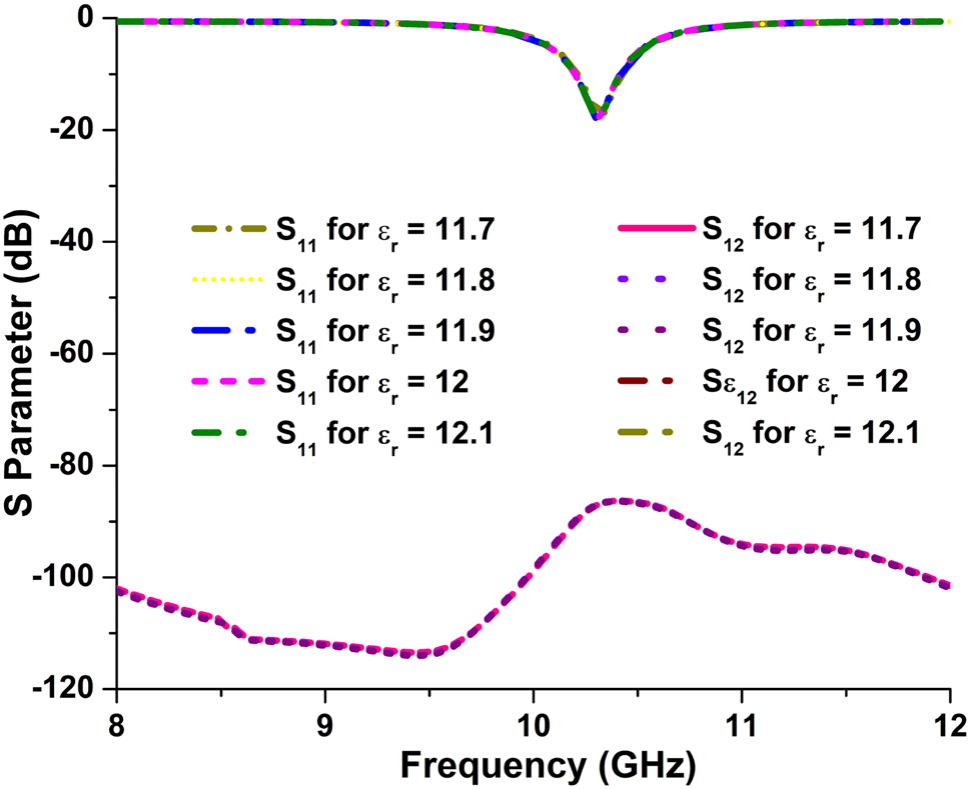
$S_{11}$ and $S_{12}$ variation with $\epsilon _r$ when two identical OCAs are placed at ‘d’ = 6 mm at 0° with x-axis.

## Parametric analysis

A detailed parametric analysis is performed to design the optimal OCA in order to achieve better performance with low interference from electromagnetic waves in the system. This analysis focuses on the impact of some of the important design parameters on the coupling behaviors of the antenna. The impact of separation distance (‘d’ ) between adjacent blocks, the $S_{12}$, and antenna gain is determined. The effect of substrate properties, mainly its dielectric constant, on the crosstalk ($S_{12}$) coefficient is also investigated. All of these analysis steps will lead to the determination of an optimum set of values for the considered parameters that provide high isolation capability as well as good gain performance of the antenna.

### $S_{12}$ versus oxide thickness

To evaluate the impact of oxide thickness on crosstalk performance, the variation of $S_{12}$ with $SiO_{2}$ thickness (‘h’) is investigated. In this study, ‘h’ is varied from 7 μm to 19 μm, while the OCA is considered without the DGS. As depicted in Fig. 5, an increase in ‘h’ results in improved isolation performance. Specifically, S$_{12}$ improves from − 32 dB to − 43 dB as ‘h’ increases from 7 μm to 19 μm, yielding an enhancement of approximately 11 dB. This improvement is attributed to the increased separation between the antenna structure and the lossy silicon substrate due to the thicker dielectric layer. In standard 180 nm CMOS technology, 9 to 11 metal layers are typically stacked with interlayer oxide thicknesses of about 2 to 3 μm^12^. However, prior studies indicate that a larger effective oxide thickness can be achieved by utilizing multiple metal layers in OCA design, thereby enhancing isolation performance^32^. Considering foundry constraints as well as the above analysis, an optimal oxide thickness of 13 μm is selected for prototyping.

### $S_{12}$ variation with $\epsilon _r$

The material used in this study is Si, which has a dielectric constant ($\epsilon _r$) of 11.9. To ensure material compatibility, the $S_{11}$ and $S_{12}$ parameters were analyzed for different values of dielectric constant ($\epsilon _r$), as shown in Fig.6. It is observed that there is negligible variation in the values of $S_{11}$ and $S_{12}$ with changing dielectric constant. Therefore, the material used for the design and simulation of the proposed structure is compatible with commonly available materials in the market, and minor variations in dielectric properties do not significantly affect the performance.

### $S_{12}$ variation with ‘d’

**Fig. 7:**
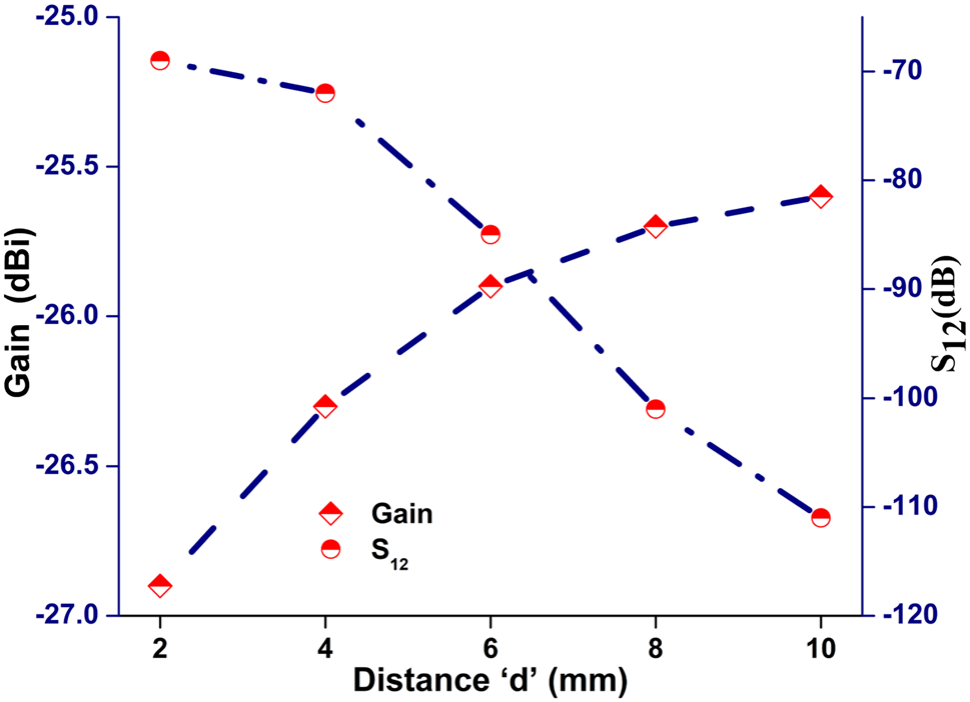
Variation in Gain and $S_{12}$ with distance between two blocks placed in 0° with x-axis.

To obtain the optimum separation between two blocks for performing the study on crosstalk, a parametric analysis is performed by varying the separation distance ‘d’.It can be seen in Fig.7 that the $S_{12}$ value gradually changes from − 69 dB to − 71 dB when ‘d’ varies from 2 mm to 4 mm. However, this reduces drastically from − 71 dB to − 85 dB for a variation of ‘d’ from 4 mm to 6 mm. The results show a monotonic improvement in isolation with increasing d (e.g., from approximately − 85 dB to − 111 dB over the investigated range of ‘d’ = 6 to 10 mm), confirming that ‘d’ is a sensitive parameter governing mutual coupling.

**Fig. 8:**
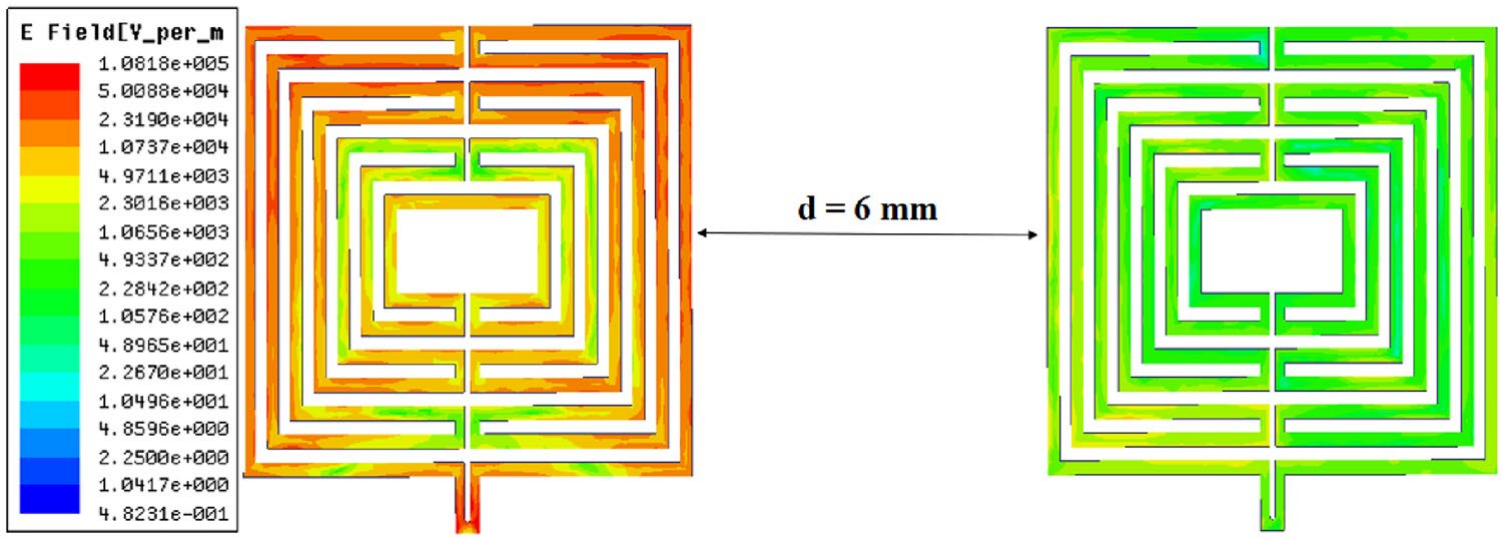
E- Field distribution to analyze the impact of crosstalk.

The impact of crosstalk on the neighbouring block, which is separated by 6 mm, is displayed through the electric field distribution in Fig. 8. In this plot, the two blocks arranged at 0°along the x-axis (i.e., the antennas are placed side by side) indicate that the induced field on the adjacent OCA decreases significantly with increasing separation distance. This behavior occurs because, when two OCAs (or nearby RF blocks) are placed in proximity, a strong parasitic capacitance exists between them. This parasitic capacitance provides an unintended coupling path, allowing high-frequency signals to transfer from one antenna to the other, thereby increasing crosstalk. As the separation distance increases, the parasitic capacitance decreases due to its inverse relationship with distance, resulting in reduced crosstalk. At ‘d’ = 6 mm, the current induced on the non-excited OCA is minimal, confirming effective isolation and reduced mutual coupling between the antennas.

### Gain variation with ‘d’

To determine the optimum value of ‘d’ , it is essential to analyze the variation in the gain of the OCA as a function of ‘d’ . This study provides important insight into the selection of ‘d’ , ensuring that it is neither too small, thereby degrading the antenna radiation performance, nor excessively large, which would violate chip area constraints. The gain variation shown in Fig.7 corresponds to a single antenna in the presence of a non-radiating component placed at a distance of ‘d’. It is observed that variation in ‘d’ predominantly affects the mutual coupling ($S_{12}$) while having a negligible impact on the radiation characteristics of individual antenna elements. The results indicate that the gain is not significantly affected as ‘d’ varies from 2 mm to 10 mm, with values ranging from − 26.8 dBi to − 25.62 dBi. This provides further flexibility in choosing ‘d’ for different applications without compromising single-element performance. However, based on parametric analysis of the gain and $S_{12}$ variation with ‘d’, an intermediate value of ‘d’ = 6 mm is selected for the fabrication of the final design, achieving a balance between radiation performance and layout constraints.

**Fig. 9:**
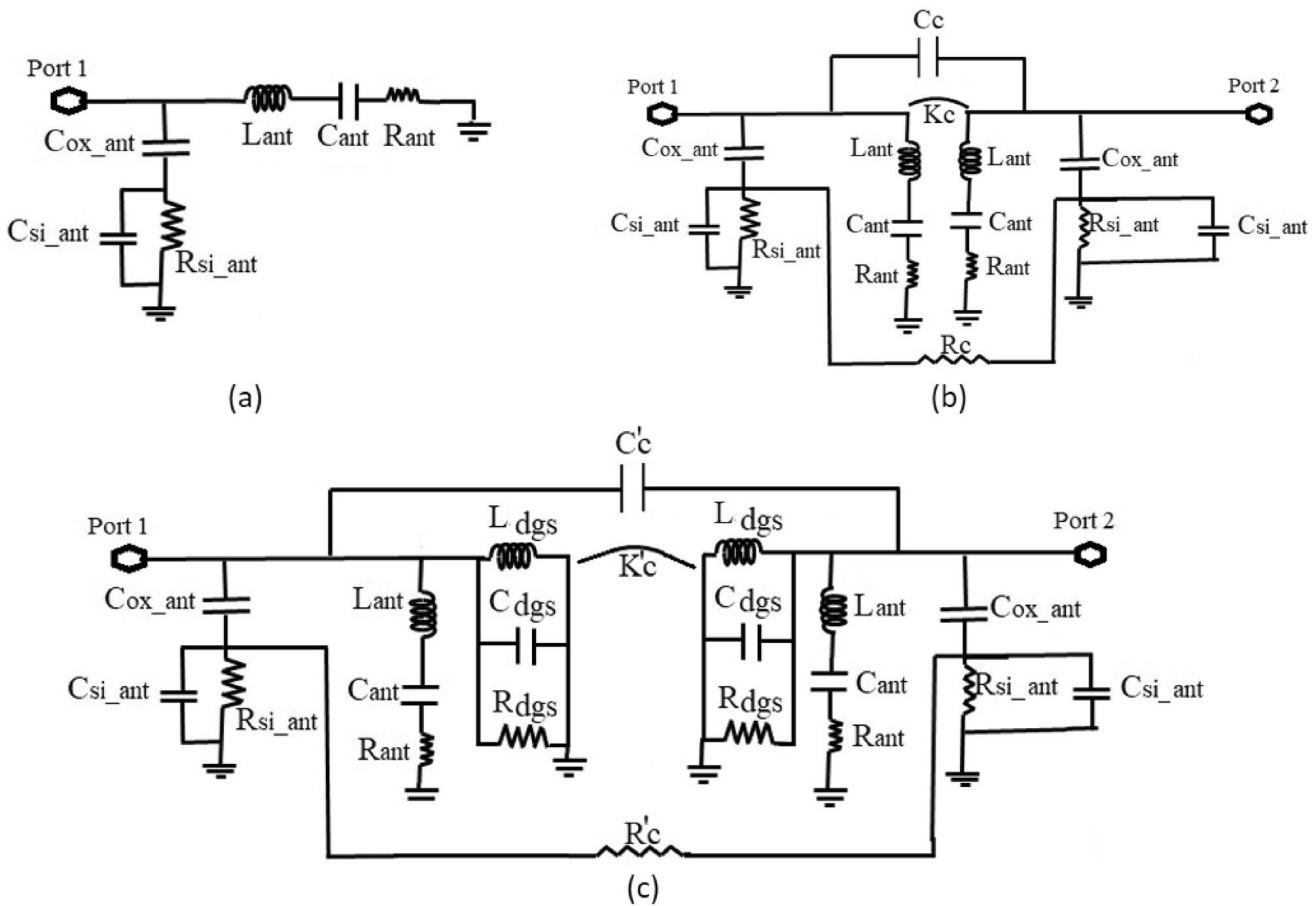
Equivalent Circuit of; (**a**) Simple On-chip Antenna; (**b**) Two adjacent OCAs; (**c**) Two adjacent OCA with DGS.

## Equivalent circuit model (ECM) for crosstalk analysis

Crosstalk is a major concern in OCA as it significantly affects the overall performance of system-on-chip (SoC) platforms in which the OCA and baseband circuits operate simultaneously. Two primary coupling mechanisms exist in OCA systems: airborne (through-the-air) coupling and substrate coupling. As Si is a lossy substrate, primarily, the surface wave mechanism can degrade OCA and the nearby blocks performance. In the proposed work, a DGS with a thick $SiO_2$ layer is employed to reduce crosstalk induced due to surface waves in OCA. The DGS layer functions as a shielding layer that suppresses electromagnetic waves propagating toward the substrate, thereby enhancing antenna performance and effectively reducing crosstalk.

To understand the coupling effect and the impact of DGS, equivalent circuits of the model have been analysed. Fig. 9a depicts the equivalent models of a simple OCA designed on an oxidized Si wafer. The antenna is represented with series RLC components, namely R$_{ant}$, L$_{ant}$, and C$_{ant}$. The capacitances generated due to the SiO$_{2}$ and Si substrate are denoted as C$_{ox}$ and C$_{Si}$, respectively. There will be a small substrate resistance R$_{Si}$, which is dependent on the resistivity of the wafer. In the proposed OCA, the resistivity of the wafer is chosen as 5 $\Omega$.cm. These two antenna circuits are kept adjacent to analyse the coupling or crosstalk effect as shown in Fig. 9b. Here, the proximity of the two OCA produces the coupling capacitance ($C_c$) and coupling resistance ($R_c$) in between. The magnetic coupling coefficient $K_c$ estimates the magnetic coupling between the OCA blocks. The $S_{12}$ is proportional to the magnitude of the magnetic coupling coefficient $K_c$, which is a prime parameter enhancing the crosstalk. To reduce the crosstalk, a DGS is incorporated in one of the metal layers of the standard CMOS layout considered for the design of the OCA. The ECM for the two DGS incorporated OCA is presented in Fig. 9c. The ECM allows for a definite qualitative explanation of how $S_{12}$ depends on the major coupling modes in operation between the two OCAs. The direct coupling mechanism via the electric field can be considered as the coupling capacitance $C'_c$ (where $C'_c < C_c$), whose role becomes pronounced at high frequencies due to a decrease in its reactance and, therefore, an increment in $S_{12}$. Concurrently, magnetic coupling can be accounted for by the mutual coupling factor Kc between the inductive elements of the DGS branches ($L_{dgs}$), giving rise to a frequency-dependent mode that becomes especially noticeable close to the resonance frequency of the DGS LC tanks. Specifically, each segment of the DGS circuit made up of $L_{dgs}$, $C_{dgs}$, and $R_{dgs}$ operates as a lossy resonator, which suppresses surface current propagation at its resonance frequency, thus diminishing the coupling. The resistive element $R_{dgs}$ determines the strength of the suppression effect, while the antenna self-impedance elements ($L_{ant}$, $C_{ant}$, $R_{ant}$) define the matching conditions.

Moreover, the inclusion of the substrate and ground-based elements, i.e., $C_{ox_{ant}}$, $C_{si_{ant}}$, $R_{c{si_{ant}}}$, and $R'_c$ (where $R'_c$ > $R_c$ ), accounting for leakage and non-infinite conductivity paths, ensures there is minimal leakage/lossy coupling between the antennas. Such elements reduce the degree of isolation and help to make $S_{12}$ response smoother without any unrealistic spikes. Thus, using such elements helps to understand the reasons for the presence of electric and magnetic coupling, as well as a leakage via the substrate with the variation in frequency due to the resonance of the DGS.

**Fig. 10:**
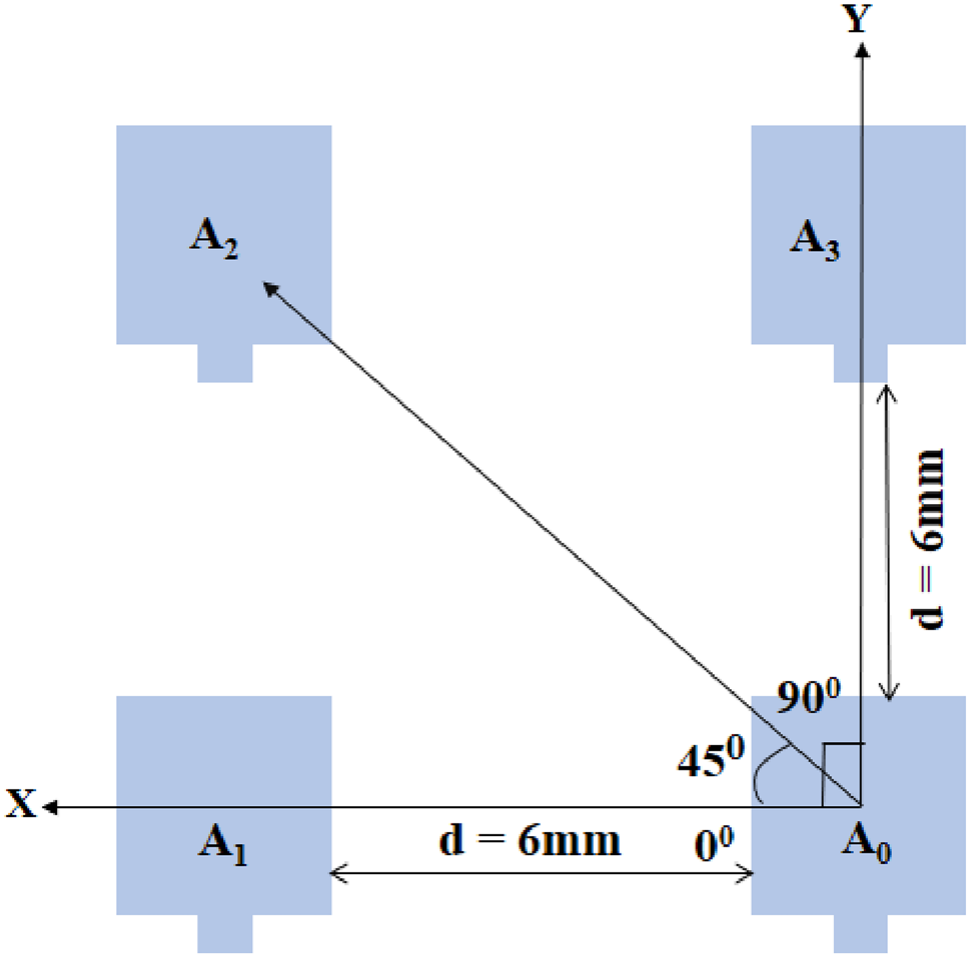
Antenna with other blocks aligned in different directions.

**Fig. 11:**
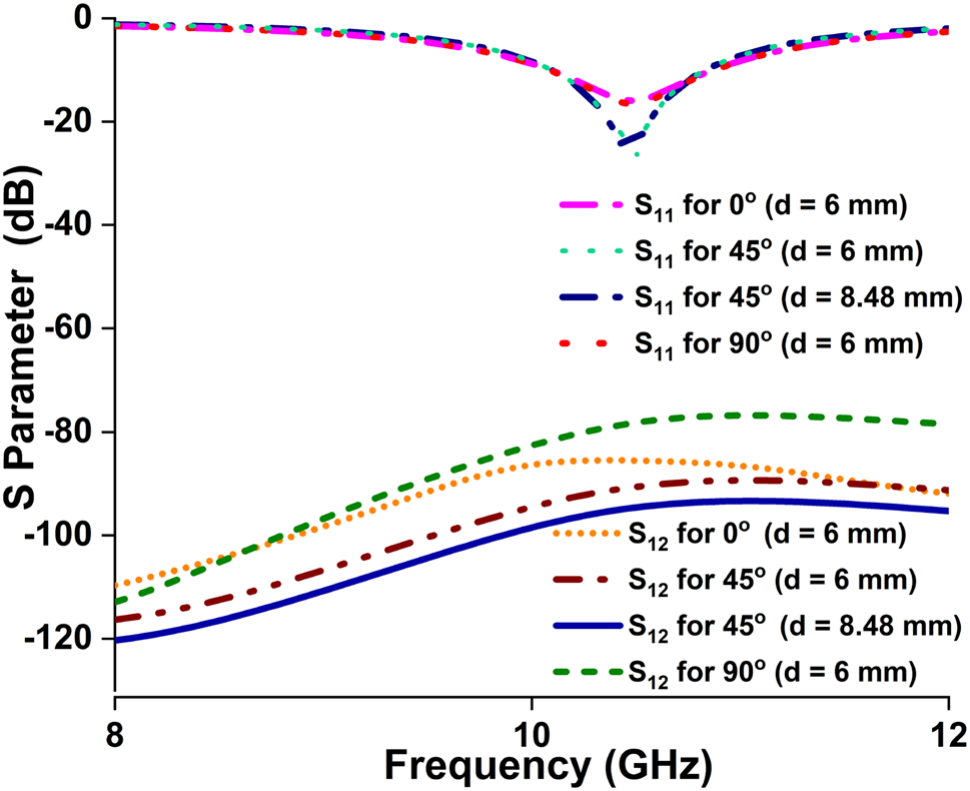
$S_{11}$ and $S_{12}$ for two OCA placed at 0°, 45°, and 90° position.

Furthermore, to analyze the crosstalk behavior based on the position of the nearby block, 3 identical OCAs are positioned at different angles with respect to a reference OCA. The reference OCA is denoted as $A_0$, while $A_1$, $A_2$, and $A_3$ are positioned at 0°, 45°, and 90° angles with respect to the x-axis, respectively, as shown in Fig. 10. The distance of $A_0$ from $A_1$ and $A_3$ is maintained at 6 mm, while $A_2$ is kept at 8.48 mm. Fig. 11 presents the simulated $S_{11}$ and $S_{12}$ parameters of the OCA for different angular orientations. The simulated $S_{11}$ values are − 26 dB, − 15.9 dB, and − 16.4 dB, indicating that the operating frequency of the reference antenna is unaffected, irrespective of the position of the nearby block. However, the $S_{12}$ analysis shows that, arguably, the diagonal position is the optimum one for the other RFIC block, as the values of $S_{12}$ reduce by 9 dB when the position is shifted from 0 °to 45 °. It is important to mention here that this reduction is achieved when ‘d’ is kept at 8.48 mm while this value is noticed as 6 dB when ‘d’ is 6 mm. However, the crosstalk increases again when the position is shifted from 45°to 90 °. Nevertheless, the positioning angle will depend upon the available space and the size of the RFIC.

## Result and discussion

**Fig. 12:**
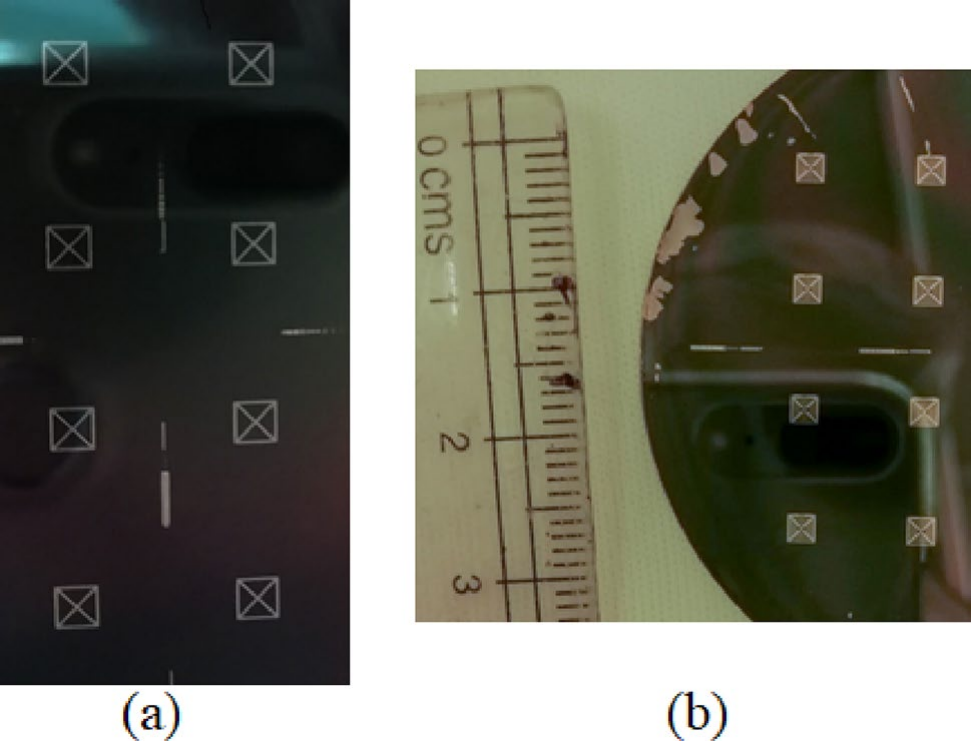
Fabricated Prototype: (**a**) Ground Plane (**b**) Top metal layer.

**Fig. 13:**
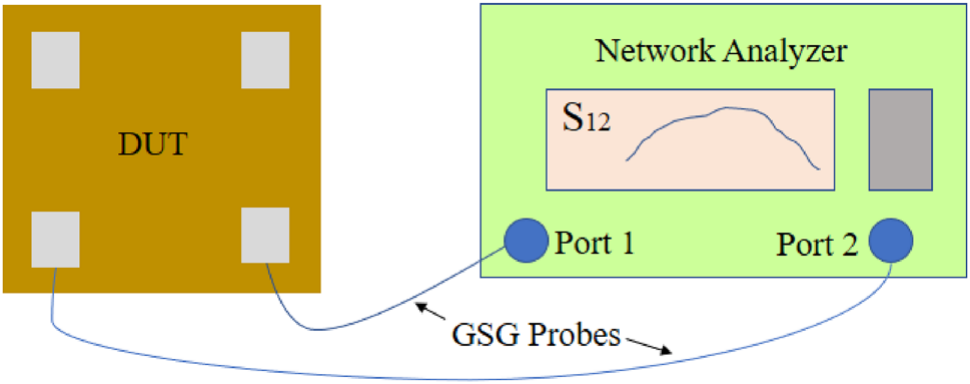
A brief illustration of the measurement setup system.

**Fig. 14:**
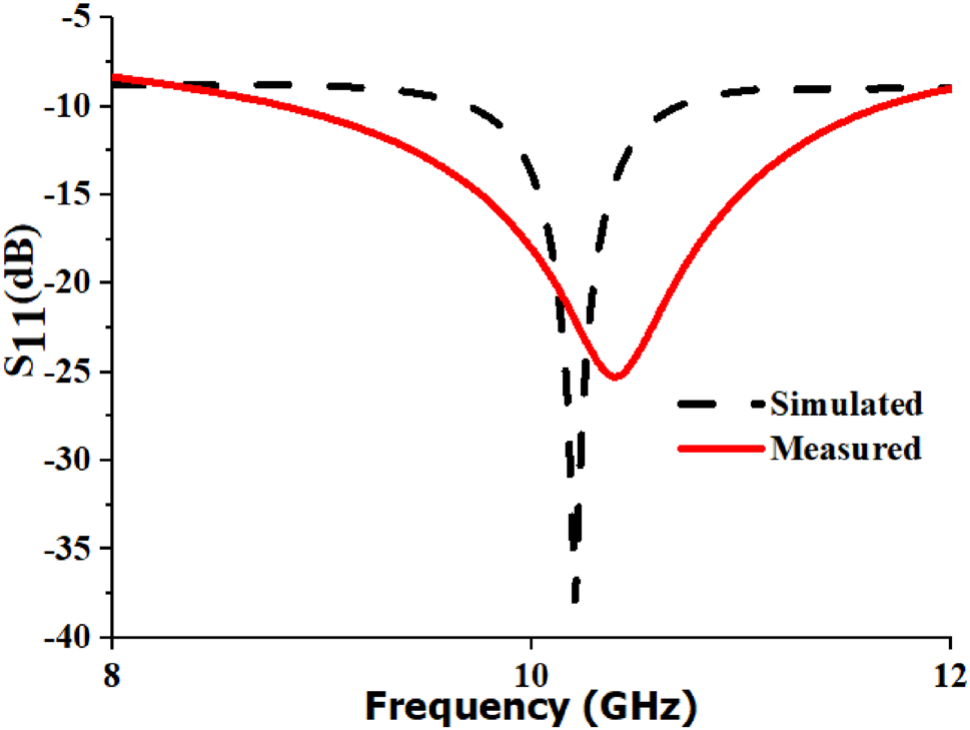
$S_{11}$ comparison with simulated and measured result of fabricated prototype.

**Fig. 15:**
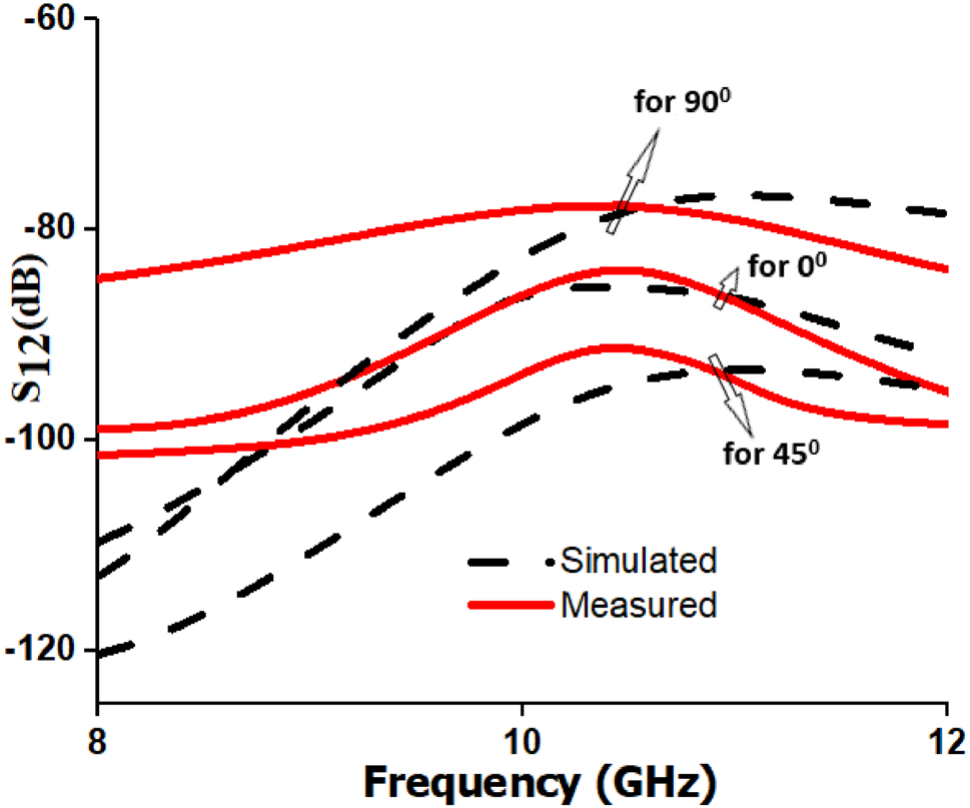
$S_{12}$ comparison with simulated and measured result of fabricated Prototype.

To evaluate the performance, the proposed meandered loop OCA is fabricated. For the fabrication process, an RCA cleaned 325 μm thick low resistivity Si wafer is used as the substrate, then a $SiO_2$ layer of thickness 3 μm is deposited over the Si wafer using the plasma enhanced chemical vapor deposition (PECVD) method. To introduce the DGS as an intermediate layer, a crossed bow-tie-shaped pattern is formed by etching 2 μm of the $SiO_2$ layer. The etching process is followed by Al metal deposition and a lift-off process to define the metal pattern and remove excess metal, respectively. As the wafer size is large, the 8 OCA prototypes were populated on the wafer. The deposited on-chip DGS ground layer is shown in Fig. 12a. Further, another layer of SiO$_2$ of thickness 10 μm is deposited by using PECVD. Subsequently, a meandered loop developed at the top layer by depositing an Al layer using the sputtering process with an optimized thickness of 1.5 μm, as demonstrated in Fig.12b. Along with that, through the etching process of the top Si$O_2$ layer, ground pads have been created for GSG probe contact. Here, two identical 200 μm pitch GSG probes are selected for the characterization. To measure the parameter $S_{11}$ and $S_{12}$ of the prototype, the block diagram of the setup for characterization is assembled as in Fig. 13. Initially, for the measurement, a Keysight MS46122B vector network analyzer (VNA) is fully calibrated with the Short, Open, Load, Through (SOLT) method to remove systematic errors introduced by cables, connectors, and adapters, thereby ensuring high measurement accuracy. After calibration of components is completed, the device-under-test (DUT) is set up for the $S_{11}$ and $S_{12}$ characterization.

First, the $S_{11}$ parameter is measured by exciting the OCA through a GSG probe, while the second port is terminated with a 50-$\Omega$ matched load to avoid any unwanted reflections and ensure accurate measurements. The measured result is displayed in Fig. 14 along with the simulated one. The measured and simulated values exhibit a high degree of correlation, exhibiting $S_{{11}}$ resonance at 10.3 GHz. However, the measured $S_{11}$ exhibits a broader frequency spectrum (or 10 dB bandwidth) than the simulated counterpart.

Second, the $S_{12}$ parameter is measured for two OCAs separated by a distance of 6 mm positioned at 0°in the fabricated prototype. These OCAs are connected to two ports of the network analyzer, where one OCA connected at port 1 is driven as the aggressor, while another OCA at port 2 acts as the victim. The $S_{12}$ of the OCA is evaluated as transmission coefficient between two adjacent element represented in decibels i.e. $S_{12}$ (dB) = 20 $log_{10}$ $|S_{12}|$ to its simulated counterpart, as illustrated in Fig. 15. A similar process is performed for the OCA positioned at ${45}^{\circ }$, and 90° orientations, respectively. The proposed design is experimentally validated over the frequency range of 8 to 12 GHz. It is observed that a noticeable deviation between simulated and measured $S_{12}$ results occurs at lower frequencies, where the maximum percentage deviation reaches up to 9%, 16%, and 22% for the 0°, 45°, and 90°orientations, respectively. The primary reason for this deviation can be attributed to fabrication tolerances and the presence of a metallic chuck located directly beneath the antenna under test (AUT), which forms an unintended parallel-plate structure that supports the propagation of substrate waves, leading to measurement discrepancies in $S_{11}$ and $S_{12}$. However, at the operating frequency of 10.3 GHz, the measured results display a good compromise with the simulated results with a minimum difference of 2.1 dB, 3.2 dB, and 1.1 dB for 0°, 45°, and 90°orientations, respectively, which shows the percentage deviation is less than 2%.

**Table 1:** Comparative analysis of the crosstalk reduction technique with the proposed method.

References	Module size (mm^2^)	Module’s blocks	‘d’ (mm)	Substrate	$f_{r}$ (GHz)	Crosstalk reduction technique	Gain (dBi)	$S_{12}$(dB)
^18^	$\pi r^2$ (r = $\lambda _0 / 16$)	Two TSV blocks	1.2	Si	8	Through Si Via	NA	− 31
^20^	$\lambda _0$ /50.6 x $\lambda _0$/8	Two monopole OCA	3	SiPh	12.6	Meandering	NA	− 25
^33^	$\lambda _0/0.42$ x $\lambda _0 /1.6$	Two microstrip Line	0.5	FR4	3.5	Circular shape DGS	NA	− 45
^34^	$\lambda _0$ /32 x $\lambda _0$/13	Two folded monopole	250	TR HR SOI	6.2	High resistivity	NA	− 44
^35^	$\lambda _0$ /2 x $\lambda _0$/3	Two microstrip patch antenna	4	FR4	3.83	EMM	NA	− 28.6
^36^	$\lambda _0$ /4 x $\lambda _0$/4	Two microstrip patch antenna	15	FR4	5	SCSRR	5	− 34
This work	$\lambda _0$ /15 x $\lambda _0$/15	Two OCA	6	Si	10.3	Meandering with DGS	− 25.9	− 85 (0°with x-axis) − 91 (45°with x-axis) − 77 (90°with x-axis)
This work	$\lambda _0$ /15 x $\lambda _0$/15	Two OCA	8.48	Si	10.3	Meandering with DGS	− 25.9	− 94

## Comparative analysis

The performance of the proposed work is compared with previously reported techniques, as summarized in Table 1. The table presents the module size, where each module has two blocks. The crosstalk analysis is performed between these two blocks. The separation distance of the blocks is separately mentioned in the table along with the nature (i.e., radiating and non-radiating) of these blocks. Several approaches have been investigated to reduce the crosstalk effect due to the antenna on the other block and vice versa. In^18,20,33,35,36^, the DGS, Electric metamaterials (EMM), Slotted Complementary Split Ring Resonators (SCSRR), TSV, and meandering techniques have been successfully implemented to reduce the crosstalk to − 45 dB, − 28.6 dB, − 34 dB, − 31 dB, and − 25 dB, respectively. Here, EMM, CSRR, DGS, and TSV are effective at minimizing mutual coupling due to the band-stop nature, but their mechanisms of action vary significantly. EMMs minimize the propagation of EM waves using negative material parameters such as negative permittivity ($\epsilon _r < 0$) or negative permeability ($\mu _r < 0$). However, CSRR, DGS, TSV, and meandering techniques suppress electric field coupling and surface current distribution through resonant disturbances. The technique, like Trap Rich High Resistivity SOI (TR HR SOI) substrate^34^ reduces the crosstalk up to − 44  dB by extending isolation between the top layer and the substrate. The enhanced performance of the proposed method can be attributed to the combined use of meandering and DGS techniques, which introduce multiple suppression mechanisms simultaneously and provide an $S_{12}$ of − 94 dB. Furthermore, this crosstalk reduction is achieved with a compact electrical size of $\lambda _0$/15 $\times$ $\lambda _0$/15 at 10.3 GHz, demonstrating the highest isolation-to-size efficiency among all compared techniques.

If compares the figure of merit which can be defined as FoM = $\frac{|S_{12}|}{ Area (Normalized)}$, the normalized FoM for the proposed design is 21150, whereas the FoM calculated for OCA^20^ and HR SOI^34^ are 10121 and 1833, respectively, and the FoM values of other reported works are significantly lower. In comparison with the second best, the proposed work demonstrates 2.09 times better FoM, which reflects that the highest isolation per unit area is achieved in the proposed design, and displays the high suppression efficiency. Notably, all the crosstalk values presented in the table correspond exclusively to the resonance frequency.

Altogether, the proposed multi-path suppression of electromagnetic coupling technique achieves ultra-high isolation, compact size, and better FoM, which promises its potential in future integrated radio-frequency systems.

## Conclusion

In this work, a study on crosstalk between two RFIC blocks is successfully performed at an operating frequency of 10.3 GHz. To deal with the worst-case scenario, both blocks are selected as OCAs. The proposed DGS-based technique is introduced in the bottom-most layer of the standard 180 nm CMOS technology node layout as the ground layer. This layer not only reduced the crosstalk but also enhanced the radiation performance of the antenna successfully. The proposed meandered loop OCA achieves significant crosstalk reduction of up to − 85 dB, − 91 dB, and − 77 dB for different orientations of the components. The regularities found in all of the performed parametric investigations suggest that the developed approach can be used not just for one particular case of operation but for any similar on-chip antennas due to the predictable nature of the electromagnetic processes involved. To understand the operating principle of DGS, an equivalent model is analyzed. Further, the crosstalk performance is evaluated for various relative positions to make this study more suitable for compact chip applications. The study suggests that a diagonal position provides the best crosstalk suppression up to − 94 dB. The fabricated prototype characterization shows good agreement between measured and simulated results. Overall, the meandered loop OCA with DGS offers an effective solution for crosstalk reduction in RFIC environments.

## Additional information

Correspondence and requests for materials should be addressed to H.S.

## Data Availability

No datasets were generated or analysed during the current study.
